# Selective interaction between phytomediated anionic silver nanoparticles and mercury leading to amalgam formation enables highly sensitive, colorimetric and memristor-based detection of mercury

**DOI:** 10.1038/s41598-020-58844-4

**Published:** 2020-02-06

**Authors:** Geetanjali M. Sangaonkar, Megha P. Desai, Tukaram D. Dongale, Kiran D. Pawar

**Affiliations:** School of Nanoscience and Biotechnology, Shivaji University, Vidyanagar, Kolhapur, Maharashtra 416004 India

**Keywords:** Biotechnology, Environmental sciences, Materials science, Nanoscience and technology

## Abstract

Presently, nanotechnology is being foreseen to play an important role in developing analytical assays for the detection of pollutants like mercury (Hg^2+^). In this study, Kokum fruit mediated silver nanoparticles (AgNPs) were differentially centrifuged to prepare anionic, monodispersed AgNPs to develop a highly sensitive, colorimetric and memristor-based assay for detection of Hg^2+^ in water samples. The investigation of the highly selective reaction between AgNPs and Hg^2+^ using HAADF-STEM images and EDS spectrum indicated the amalgam formation through etching and under potential deposition which resulted in a visible color change from brown to colorless, change in SPR intensity and also change in memristive switching like property of AgNPs. The developed colorimetric assay detected Hg^2+^ with a limit of detection (LOD) of 6.2 ppb and limit of quantification (LOQ) of 18.9 ppb and, quantitatively recovered Hg^2+^ with good accuracy and precision (RSD < 2%). Further, the test of memristive switching like property of AgNPs demonstrated frequency-dependent shrinkage of I–V hysteresis loop indicating memristive switching like property. The test of the sensitivity of Hg^2+^ detection was estimated to be 8.7 ppb as the LOD and 26.4 ppb as LOQ. Like the colorimetric assay, the memristor-based assay also recovered Hg^2+^ with good accuracy and precision.

## Introduction

Residues of mercury (Hg) based organic and inorganic compounds that are used as pesticide or fungicides in agriculture practices can accumulate in aquatic organisms. The residual toxicity of Hg can lead to serious health issues in animals and humans who consume such contaminated aquatic organisms^1^. Coal-burning power plants are the main sources of the pollution of Hg. In addition, other sources from natural and human activities are also known to contribute to Hg pollution^2,3^. It is known that water dissolved ions of mercury (Hg^2+^) are the most common and stable form of Hg for which point of care detection is necessary to detect and monitor the Hg^2+^ levels. Devising selective and sensitive methods for point of care detection of Hg^2+^ from clinical diagnostics, household, environment, and industrial waste samples is expected to greatly help protect public health and the environment^4^.

Recently, nanotechnology has been foreseen to play a significant and important role in developing the lab-on-chip devices for the detection of environmental pollutants. Metal nanoparticles (NPs) such a silver NPs (AgNPs) and gold NPs (AuNPs) have been demonstrated for their use in heavy metal sensing application^4–8^. Recently, differently synthesized and modified AgNPs and AuNPs were demonstrated for their use in Hg^2+^ detection^9–12^. It is noteworthy that all these studies reported the need for functionalization and formation of the complex of a ligand with Hg^2+^ for detection. The procedures of surface functionalization of nanomaterials (NMs) are somewhat tedious, prone to contamination, require sophisticated instruments and trained personnel which make the on-site detection difficult^13,14^. Therefore, unmodified NMs based simple, rapid and accurate methods for the detection of Hg^2+^ would alleviate the need for functionalization and associated bottlenecks.

In this study, we have demonstrated the use of unmodified, monodispersed, anionic AgNPs for highly sensitive, colorimetric and memristor-based detection of Hg^2+^ in real water samples. To this end, the differential centrifugation technique was employed to prepare Kokum fruit extract mediated, monodispersed, biogenic AgNPs. The AgNPs were then characterized by various spectroscopic and imaging techniques such as UV-Vis spectroscopy, dynamic light scattering (DLS) with zeta potential analysis, Fourier transform infrared spectrometer (FTIR), X-ray diffractometer (XRD), energy-dispersive X-ray spectroscopy (EDS), transmission electron microscopy (TEM) and selected area electron diffraction (SAED). The highly selective detection potential of AgNPs toward selected metal ion (Hg^2+^) was investigated by studying the elemental composition of detection reaction employing high-angle annular dark-field scanning TEM (HAADF-STEM) imaging and EDS spectrum analysis. Biogenic AgNPs were then employed as a label-free, highly sensitive and selective probe for colorimetric detection of Hg^2+^ in real water samples. In addition, the memristor-based I-V switching behavior of AgNPs was studied and employed for the development of highly sensitive, precise and accurate memristor-based methods for the detection of Hg^2+^.

## Results and Discussion

### Biosynthesis and preparation of monodispersed, colloidal AgNPs

**Figure 1:**
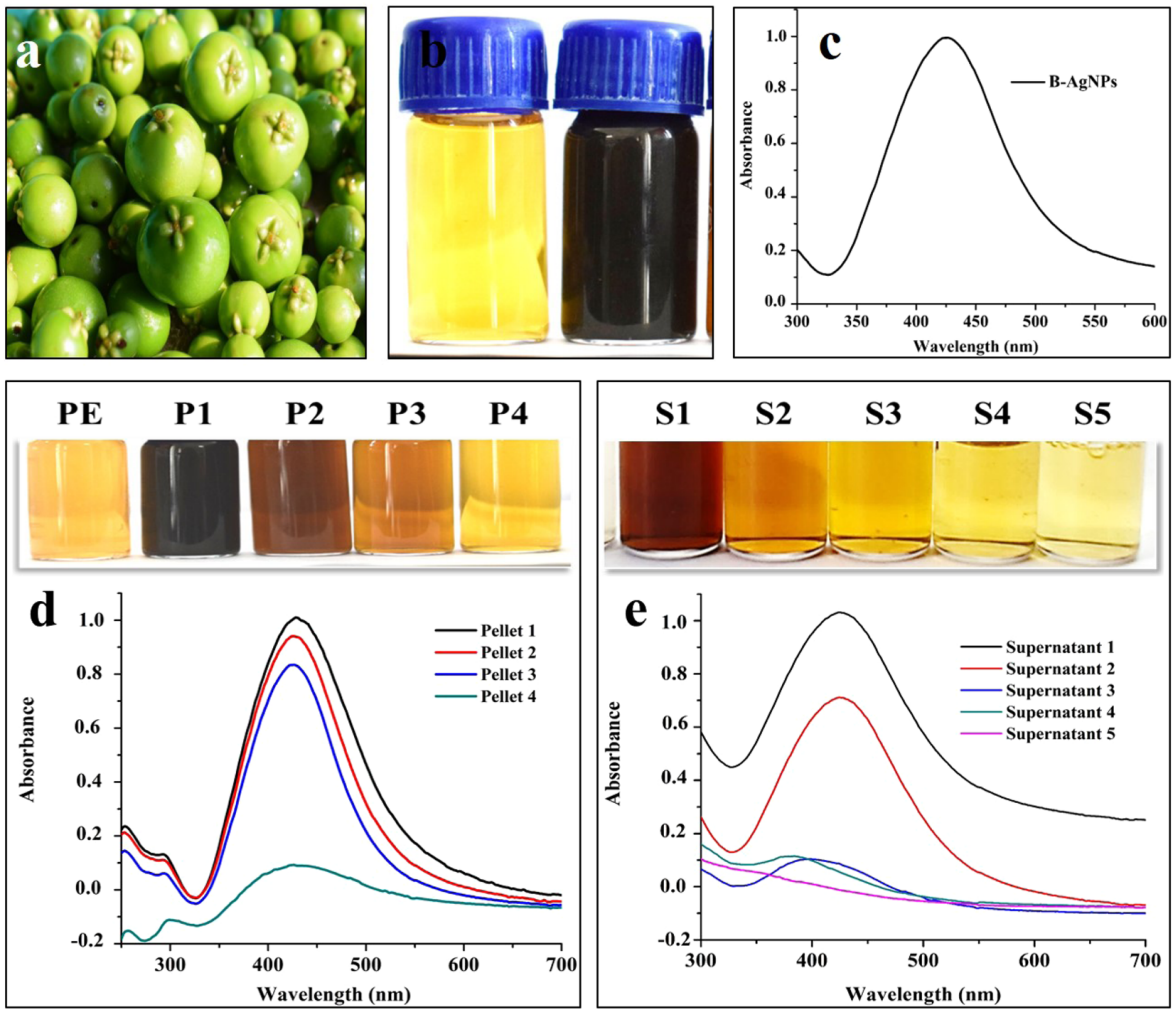
(**a**) Unripe Kokum fruits; (**b**) Kokum fruit extract and colloidal biogenic AgNPs; (**c**) UV−vis. spectra of AgNPs; (**d**) UV−Vis. spectra of colloidal AgNPs solution obtained after re-suspending pellets prepared by differential centrifugation. Inset images show colours of re-suspended pellets; (**e**) UV−vis. spectra of AgNPs in supernatants prepared by differential centrifugation. Inset images show colours of supernatant.

The biogenic synthesis reaction between Kokum fruit (Fig. 1a) extract and 1 mM AgNO_3_ at pH 8 and, incubation at 70 °C for 10 min resulted in change of color from pale yellow to dark brown (Fig. 1b) and appearance of characteristic Surface Plasmon Resonance (SPR) peak at 426 nm (Fig. 1c) which confirmed the biosynthesis of AgNPs. The initial dark brown AgNPs colloidal solution and its broad SPR peak indicated the polydispersed preparation with broad particle size distribution. For metal NPs probe-based calorimetric detection, well dispersed, colloidal preparation is mostly preferred since plasmonic response depends on their size, shape, dielectric environment and mutual electromagnetic interactions among particles in close proximity^15^. The use of differential centrifugation to reduce the polydispersity resulted in four pellets and five supernatants. When pellets 1–4 were re-suspended in sterile double distilled water (SDDW), a brown and transparent colloidal solution of AgNPs without any aggregation indicating good dispersity was formed. The color intensities of the colloidal solutions formed after re-suspension of pellets gradually decreased from pellet 1 to 4 (Fig. 1d). Likewise, in the case of obtained supernatants also, the brown color intensities were found to gradually decrease from supernatant 1 to 4 whereas, the color of supernatant 5 was found pale yellow with no characteristic SPR peak indicating the absence of AgNPs (Fig. 1e). Further, monitoring the particle size in re-suspended pellets showed a decreasing pattern of particle sizes from pellet 1 to 4. Comparatively, among all 4 pellets, AgNPs in pellet 3 which was prepared by sequential centrifugation at 15000 rpm for 15 min, were of small size, less polydispersed (Fig. S1a–d) with good absorbance intensity, therefore, were further selected, characterized and used in colorimetric and memristor-based detection of metal ions. The average zeta potential of AgNPs of pellet 3 was −20 mV that indicted anionic nature (negative charge) due to the presence of capped anions on the surface and good stability in the colloidal state (Fig. S1e).

### Characterization of phytomediated AgNPs

**Figure 2:**
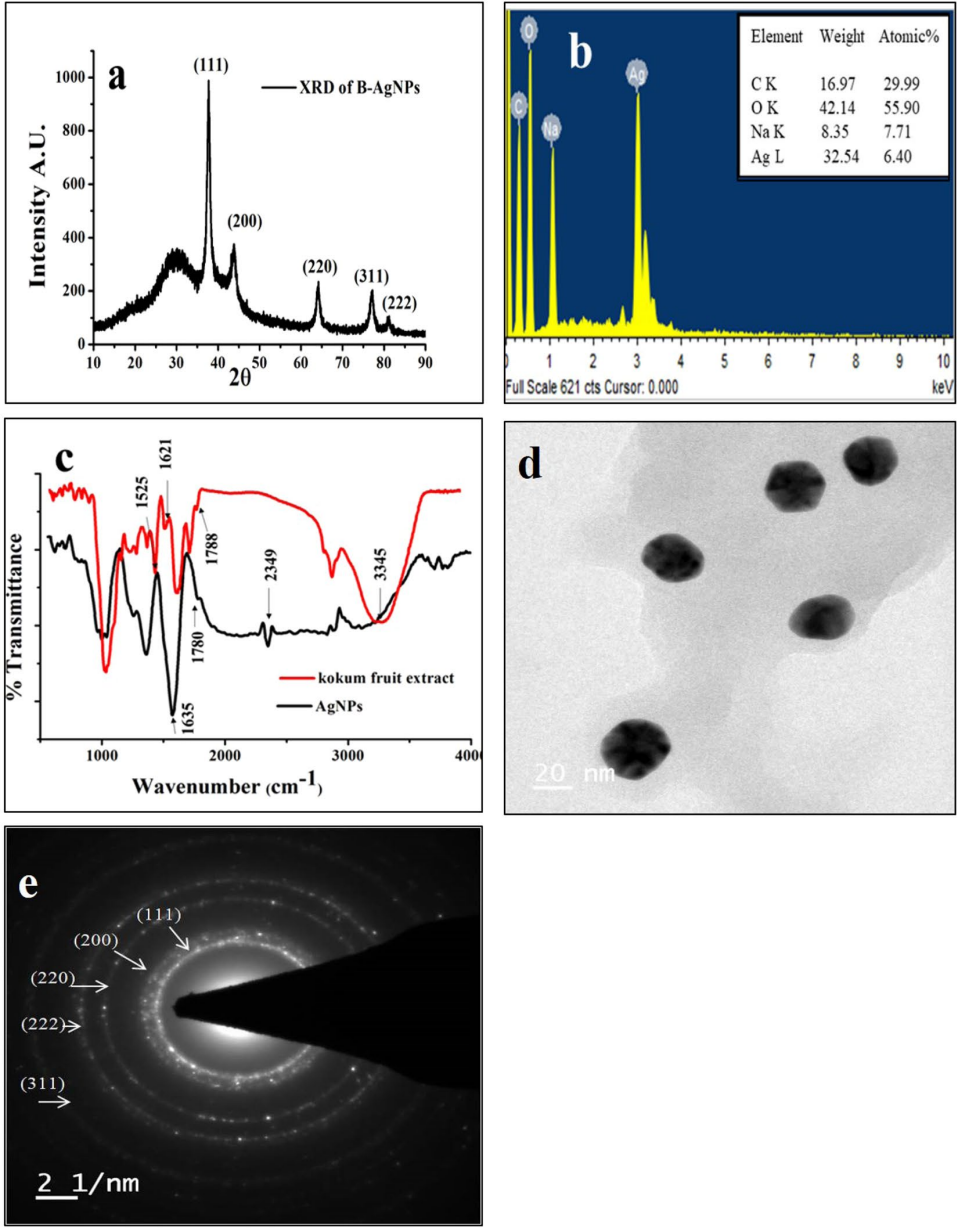
(**a**) XRD pattern of AgNPs; (**b**) EDS spectrum of AgNPs; (**c**) FTIR spectra of AgNPs and Kokum fruit extract; (**d**) TEM image of AgNPs (**e**) SAED pattern of AgNPs.

The analysis of XRD pattern of AgNPs of pellet 3 showed 2θ values of 38.101, 44.370, 64.179, 77.549, 81.506 representing (111), (200), (220), (311) and (222) planes, respectively (Fig. 2a). The crystallographic parameters such as crystal system: Cubic, Space group: Fm-3m; Space group number: 225 indicated that AgNPs were nanocrystals with cubic face-centered (FCC) structure. The peaks in the XRD pattern confirmed that NPs of the pellet 3 were pure AgNPs with highly crystalline nature. The pattern was consistent and matched with the database Joint Committee on Powder Diffraction Standards (JCPDS) card No. 00-001-1167^16–19^. The study of elemental composition by EDS pattern showed sharp peaks of carbon, oxygen, and silver at 3 keV that confirmed the presence of crystalline AgNPs of biogenic nature (Fig. 2b).

Recording and comparing the FTIR spectra of Kokum fruit extract and AgNPs (Fig. 2c) showed the local surface structure and possible functional groups of biomolecules that could have involved in the reduction and stability of AgNPs. In the FTIR spectrum of fruit extract, a peak corresponding to vibrational stretching in the region 3345 cm^−1^ could be attributed to OH stretching and presence of −NH_2_ and −OH groups from an amino acid. The absorption peak at 2349 cm^−1^ in the FTIR spectrum of AgNPs represented thiol groups of amino acid^20^ whereas peaks at 1788 and 1780 cm^−1^ in the FTIR spectra of extract and AgNPs respectively could be attributed to stretching vibrations of CO functional groups of aldehydes, ketones, and carboxylic acids. The IR peaks at 1621 and 1525 cm^−1^ in the spectrum of extract and corresponding peaks at 1635 cm^−1^ in the spectrum of AgNPs were attributed to the stretching vibration of N-H and the presence of amide I and II. As reported earlier, these peaks could have arisen due to the contribution of −NH_2_ and −COO− groups to the reduction and stabilization of AgNPs^21^. The broad band observed around 3400 cm^−1^ in the fruit extract may be due to the existence of water molecules. These FTIR based observations suggested the involvement of −NH_2_ and −COO− functional groups of amino acids of Kokum fruit extract in the biogenic synthesis of AgNPs (Fig. 2c). The imaging of AgNPs by TEM showed the overall quasi-spherical morphology with a mostly spherical and polygonal shape with minimum polydispersity in the size range of 22–25 nm (Fig. 2d). Further, analysis by SAED pattern exhibited different phase arrangements of five circular bright rings corresponding to the (111), (200), (220), and (311) and (222) planes indexed to FCC crystalline structure of AgNPs (Fig. 2e). The crystalline structure of AgNPs demonstrated by SAED was in congruence with the XRD pattern.

### Screening of metallic cations for the test of selectivity of colorimetric detection

**Figure 3:**
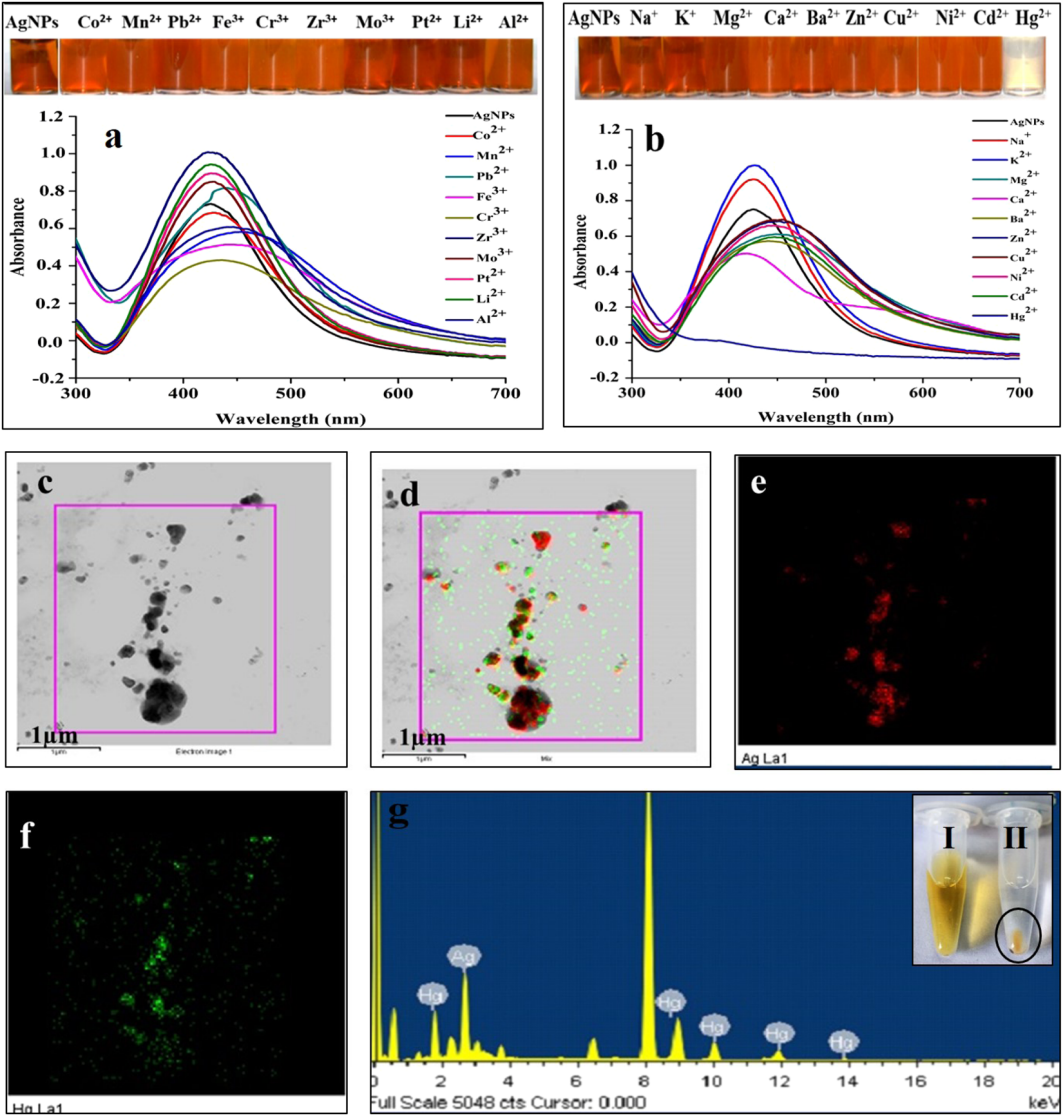
(**a**,**b**) UV-vis. absorption spectra and change of reaction colours (inset) for selective screening of AgNPs probe based detection of 20 different metallic cations; (**c**) HAADF-STEM image of amalgam formed between AgNP and Hg^2+^; (**d**) EDS mapping of overlapped elements; (**e**) EDS mapping of Ag; (**f**) EDS mapping of Hg and (**g**) EDS spectrum of the plotted area. Inset image shows (I) solution of AgNPs and (II) amalgam formed between AgNP and Hg^2+^ in detection reaction.

The visible disappearance of brown color and change in SPR intensity were not observed when alkali metals such as Li^+^, Na^+^, K^+^, alkaline earth metals like Mg^2+^, Ca^2+^, Ba^2+^ and transition metals like Zn^3+^, Cu^2+^, Ni^2+^, Cd^2+^, Hg^2+^, Co^2+^, Mn^2+^, Pb^2+^, Fe^3+^, Cr^3+^, Zr^3+^, Mo^3+^, Pt^3+^ were reacted with AgNPs solution. However, when Hg^2+^ was added, a remarkable visible color change from brown to colorless and change in SPR intensity was observed (Fig. 3a,b). This interaction AgNPs with Hg^2+^ clearly demonstrated very high selectivity and specificity toward colorimetric detection of Hg^2+^ whereas, AgNPs were not found sensitive to other tested cations under similar assay conditions. Further, monitoring the colorimetric responses of the detection reactions through UV-Vis. spectroscopy showed specific spectral shifts and slight decreases in SPR intensities for all tested metal cations, but in the case of Hg^2+^ ions, SPR intensity was significantly decreased and reached to zero (Fig. 3b). Since, in our study, no significant changes in color, extinction maxima, and SPR intensities were observed upon interactions of AgNPs with all tested cations except Hg^2+^, the assay approach showed very high selectivity toward colorimetric detection of Hg^2+^.

Recently, two possible mechanisms were proposed and implicated for observed decreased absorbance and SPR shift upon the interaction of AgNPs with Hg^2+^ ^22^. As per the first mechanism, surface coating of Hg° ions on AgNPs takes place which subsequently leads to decreased absorbance and shift in SPR, whereas, the second mechanism advocates the formation of amalgam between AgNPs and Hg ions. Amalgam formation seems to be the most likely mechanism as the electrochemical potentials difference between Hg^2+^(0.85 V) and AgNPs (0.8 V) is very small which makes Hg^2+^ and AgNPs capable of chemical interaction through under-potential deposition (UPD) leading to amalgam formation^22^. As reported by Tanvir *et al*., (2019), Rex *et al*., (2006), and Kamali *et al*., (2016), the combination of silver or gold with mercury can cause amalgam formation which leads to a blue shift of the absorption peak^23–25^ (Fig. 3b). In our case, similar marginal blue shifts in the absorption peak were also observed indicating the amalgam formation.

In order to confirm the selective colorimetric detection of Hg^2+^ through the mechanism of amalgam formation, HAADF-STEM images and EDS spectrum of detection reaction were recorded and analyzed. Figure 3c–g represent STEM-HAADF images and elemental mapping which clearly showed that amalgam was composed of AgNPs with Hg^2+^. These results demonstrated that during colorimetric detection, AgNPs selectively interacted with Hg^2+^, formed amalgam and subsequently changed the color of reaction, decrease absorbance and cause SPR shift (Fig. 3c–g)^.^ Further, in order to confirm the amalgam formation, the detection reaction AgNPs and Hg was performed and subjected to high-speed centrifugation to collect the pellet of amalgam (Fig. 3g inset image). This pellet was then washed twice, dried and analyzed by FTIR (Fig. S2). The IR peak due to the presence of amide I and II at 1635.5 cm^−1^ in the spectrum of AgNPs (Fig. 2c) was also observed in the IR spectrum of Ag-Hg amalgam. The transmittance of other peaks in the range of 1500–1700 was found changed in the spectrum of Ag-Hg amalgam. Further, the FTIR spectrum indicated that the absorption peak at 2349 cm^−1^ of thiol groups of amino acid was shifted to 2122.54 cm^−1^ which could have occurred due to the interaction of AgNPs with Hg^2+^ (Fig. S2). Similar shifting of thiol group was also reported by Aktarra *et al*.^26^.

### Colorimetric detection of Hg^2+^ in real water samples

**Figure 4:**
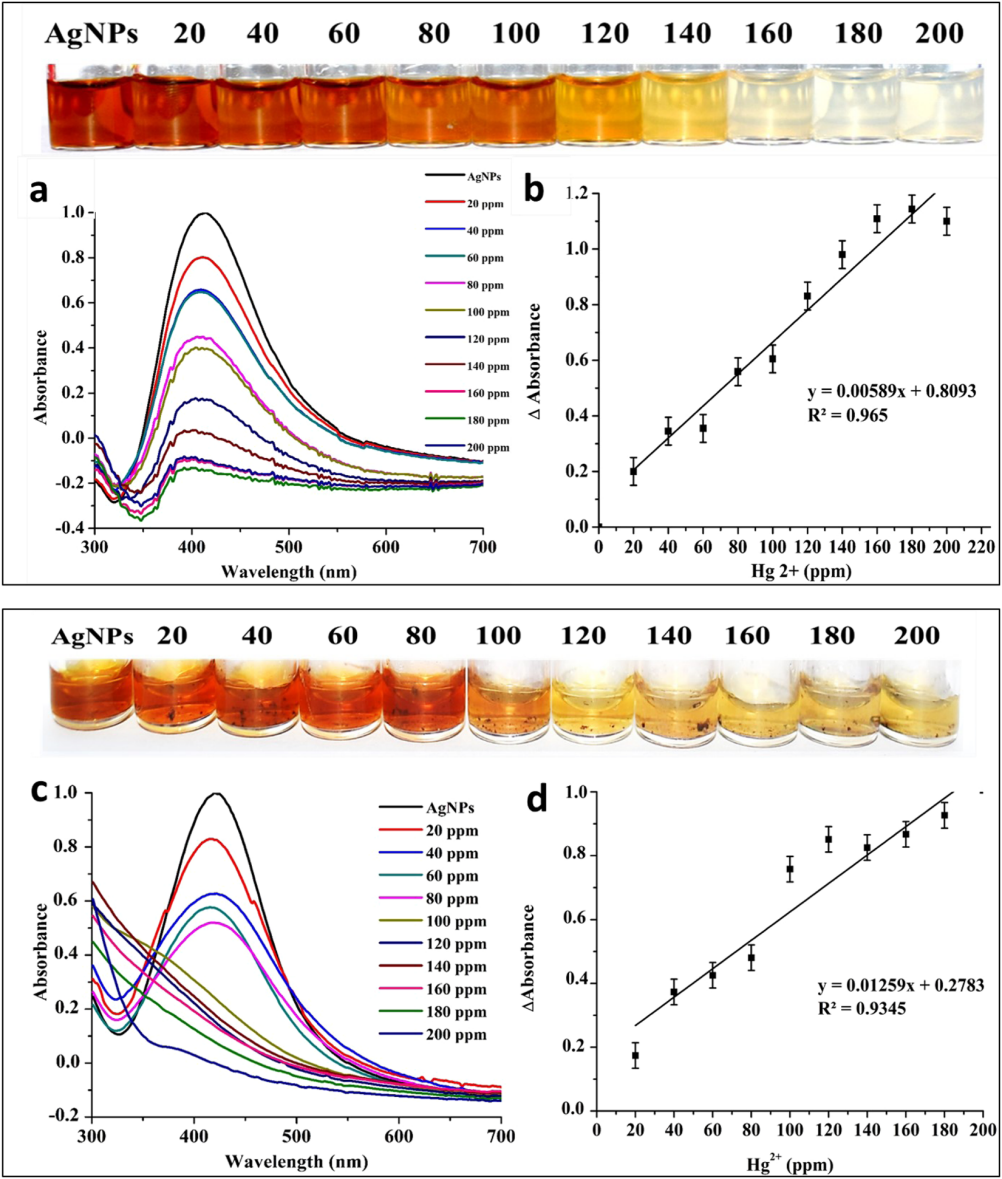
UV-Vis. absorption spectra and change of reaction colours (inset) for AgNPs probe based detection of Hg^2+^ in the range of 20–200 ppm in SDDW (**a**) and tap water (**c**); plot of ΔAbs (change in absorbance) against concentration of Hg^2+^ in SDDW (**b**) and tap water (**d**).

In the present study, the potential application of the AgNPs based colorimetric assay was demonstrated by detecting Hg^2+^ in real water samples such as SDDW (Fig. 4a,b) and tap water (Fig. 4c,d) spiked with known concentrations of Hg^2+^. Initially, color change from brown to colorless was not observed when SDDW and tap water samples spiked up to 120 ppm of Hg^2+^ were added, whereas, the color intensity gradually decreased by addition Hg^2+^ in the range of 120–200 ppm. The complete decoloration of the AgNPs solution was observed upon the addition of both samples with 200 ppm of Hg^2+^ (Fig. 4a,c). The resulting change in color due to corresponding change in SPR properties of AgNPs with increased Hg^2+^ concentration was clearly visible to naked eyes. To explain the mechanism of decoloration, a recent work by Narayanan and Han 2017 proposed the redox reaction equation and attributed the decoloration and formation of whitish color to the release of Ag^+^ ions and their subsequent reaction with Cl^−^ ions to form AgCl_2_^27^_._ Likewise, in congruence with the mechanism proposed by Manivannan *et al*. 2018, Farhadi and coworkers demonstrated that the decoloration takes place due to etching of AgNPs by Hg^2+^as standard electrode potential of Hg^2+^/Hg (0.85 V) is slightly higher than that of the Ag^2+^/Ag (0.80 V). This causes redox etching of AgNPs due to the deposition of Hg^0^ on the etched AgNPs by means of amalgam formation leading to the disappearance of brown color^5,22^.

In our study, monitoring the UV-vis spectra of detection reactions in the range of 20–200 ppm of Hg^2+^ showed a gradual decrease in SPR intensities at wavelength 414 nm (SDDW) and 420 nm (tap water) along with a blue shift (Fig. 4b–d). The SPR intensities reached to zero upon addition of samples with 120 and 100 ppm of Hg^2+^ in SDDW and tap water, respectively. The plot of Hg^2+^ concentration Vs Δabs indicated the linear relationship with linear regression coefficient (R^2^) of 0.9744 and 0.9653, LOD of 41.51 and 43.37 ppm, and limit of quantification LOQ of 125.78 and 131.43 ppm for tap water and SDDW samples, respectively (Fig. 4b,d). The observed minor differences in LODs and LOQs between tap water and SDDW could have occurred due to the presence of unknown dissolved matters^28^ and contaminants other than Hg^2+^ in tape water samples. Such presence of unknown contaminants in water samples is known to interfere with the detection and analysis of Hg^2+^ ions^21^.

### Test of accuracy and precision of colorimetric detection of Hg^2+^

**Figure 5:**
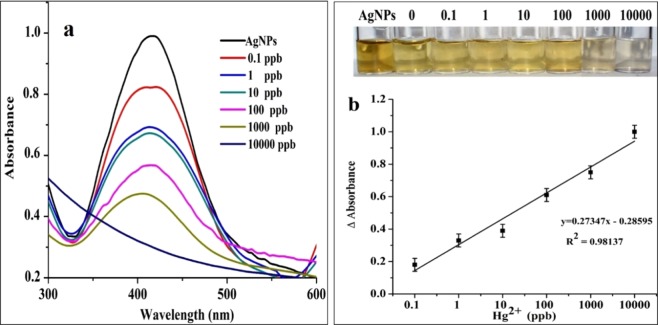
(**a**) UV-Vis. absorption spectra of AgNPs probe based Hg^2+^ detection reactions in the range of 0.1–10000 ppb in SDDW; (**b**) corresponding standard plot of ΔAbs against concentration of Hg^2+^ in SDDW (inset photograph shows gradual change in colour from yellow to colourless upon reaction of AgNPs with Hg^2+^ in the range 0.1–10,000 ppb).

In order to enhance the sensitivity of the colorimetric assay, the effect of dilution of the original AgNPs (pellet 3) stock solution on sensitivity was studied. To this end, the stock solution was diluted five times and employed for the detection of Hg^2+^ in SDDW in the range of 0.1–10000 ppb. The detection reactions in this tested range resulted in the recognizable change of color (Fig. 5b inset image). As shown in Fig. 5a, the SPR intensities showed a definite and reproducible decrease within five minutes, even at the lowest of 0.1 ppb of Hg^2+^. Thus, the sensitivity of the colorimetric assay was increased when the AgNPs stock solution was diluted and used. The highly sensitive colorimetric assay of the present study provided a good linear calibration range over 0.1–10000 ppb with a linear regression coefficient (R^2^) of 0.9845, LOD of 6.2 ppb and LOQ of 18.9 ppb (Fig. 5b). The estimated LOD is close to the permissible limit (2 ppb) in drinking water declared by the World Health Organization (WHO)^29^ that makes the present assay suitable for analysis of real-life samples. The use of UV-Vis spectroscopy further improved the sensitivity of the method up to 0.1 ppb. In comparison, the sensitivity of the present colorimetric assay is by far the best than previously reported biogenic AgNPs based assays (Table S1).

The % recoveries for intra-day repeatability and inter-day reproducibility indicating the accuracy and precision of colorimetric assay are shown in Table S2. From SDDW, intra-day % recoveries of Hg^2+^ were estimated in the range of 98.2–110.8 with %RSD in the range of 0.11–0.44, while inter-day % recoveries were estimated in the range of 104–113 with %RSD in the range of 0.02–0.85. Similarly, for tap water samples, intra-day % recoveries were estimated in the range of 95.73–115.2 with %RSD in the range of 0.33–0.73 while, inter-day % recoveries were estimated in the range of 100.1–118.4 with %RSD in the range of 0.15–0.89 (Table S2). As biogenic AgNPs based colorimetric assay demonstrated in the present study quantitatively recovered Hg^2+^ with good accuracy and precision (RSD < 2%), it complies with the norms laid by United States Pharmacopeia (USP)^30,31^ and therefore, is acceptable.

### Test of memristive switching property of biogenic AgNPs

Previous studies by Manivannan *et al*., 2018 and Farhadi *et al*., 2012 attributed the decoloration of the brown color of AgNPs solution to etching of AgNPs by Hg^2+^ and subsequent UPD of Hg^2+,^^5,22^. In congruence with these reports, our HAADF-STEM images and EDS spectrum analyses of colorimetric reaction (Fig. 3c–g) also confirmed the Hg^2+^ detection through amalgam formation. Therefore, we presumed that these etching and UPD leading to amalgam formation could also change memristive switching like property of AgNPs. Hence, we hypothesized that biogenic AgNPs could also be employed for highly specific and sensitive detection of Hg^2+^ based on memristive switching effect. Considered as a fourth fundamental circuit element along with resistor, capacitor, and an inductor^32^, the memristive devices are better known for their memory with resistance property. The memristive devices basically depend on two important recognition criteria such as hysteresis loop in I–V plane and frequency-dependent I-V hysteresis loop or limiting linear characteristic^33–35^. As per the theory of limiting linear characteristics, the I-V hysteresis loop shrinks as the frequency of applied signal increases. For the test of the memristive switching effect of AgNPs, a sandwich model-based detection device (Fig. S3) fabricated using the FTO substrate was best suited as FTO conducting sides functioned as metal and space between them worked as an active layer. This active layer was large enough to be filled with colloidal AgNPs and analyte to be tested.

**Figure 6:**
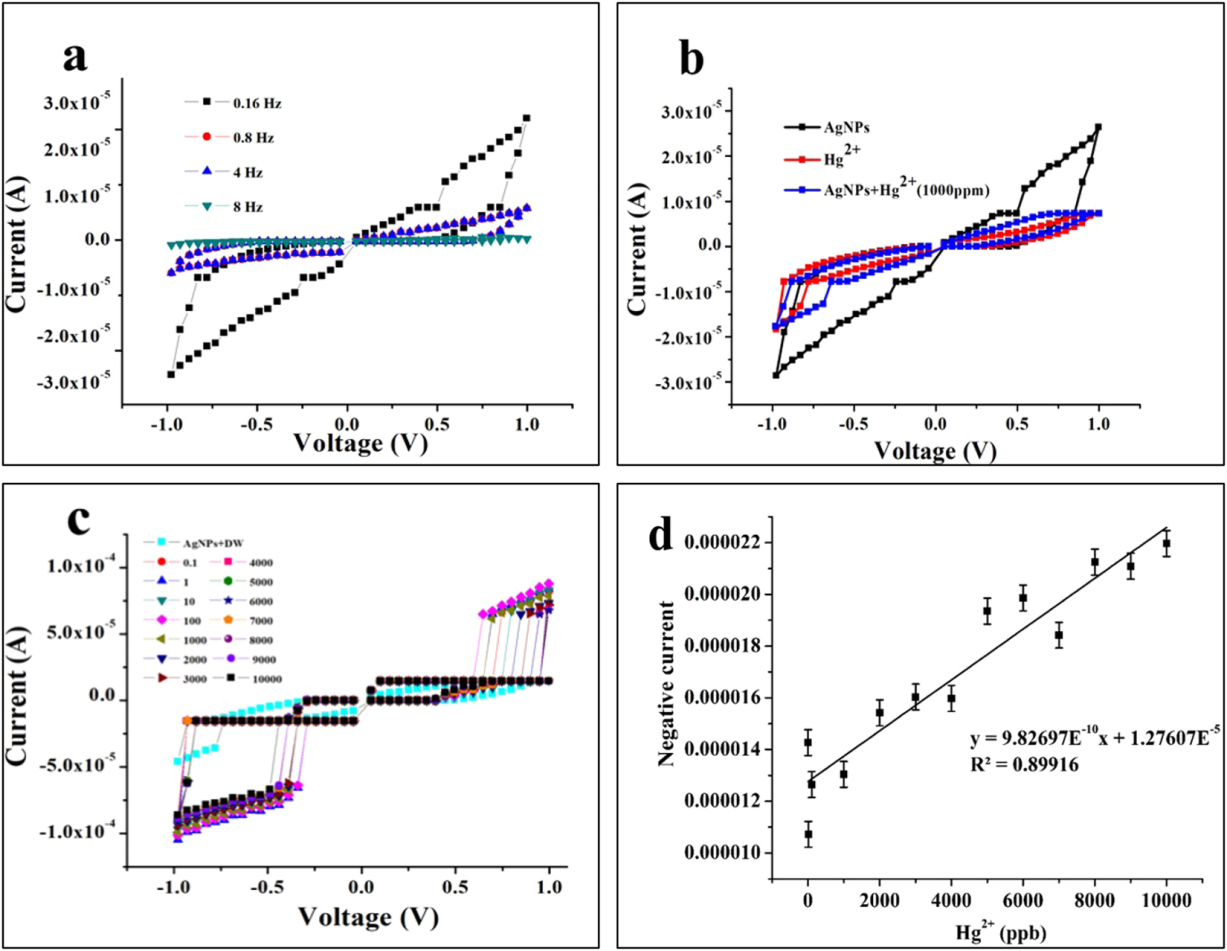
(**a**) Frequency dependent I-V switching of AgNPs; (**b**) I-V switching of AgNPs, Hg^2+^, AgNPs + Hg^2+^ at 0.16 Hz; (**c**), Hg^2+^ concentration dependant I-V switching of AgNPs at frequency 0.16 Hz; (**d**) plot of Hg^2+^ concentration against maximum negative current of AgNPs.

To test memristive switching like property, the frequency-dependent memristive switching of AgNPs was measured at fixed external voltage bias (±1 V) (Fig. 6a). This measurement clearly indicated the memristive switching like property of AgNPs which was evident by the observed hysteresis loop. In addition, frequency-dependent measurements of I–V led to shrinkage of the hysteresis loop as the frequency of the applied signal was increased (Fig. 6a). Noteworthy, good memristive switching and hysteresis loop was observed for the lowest frequency of operation (0.16 Hz). These observations demonstrated that biogenic AgNPs possessed memristive like behavior and the fabricated device performance was in accordance with the limiting linear characteristic theory of memristive device^36^.

Further, to study how the memristive like the behavior of AgNPs responds in presence of Hg^2+^, I-V switching of Hg^2+^ (1000 ppm) alone and AgNPs + Hg^2+^ (1000 ppm) at 0.16 Hz were recorded and analyzed (Fig. 6b). The measurements indicated that only AgNPs show a higher current and memristive switching area than the Hg^2+^ alone and AgNPs + Hg^2+^. It was presumed and expected that after the addition of Hg^2+^, AgNPs would show the higher current and memristive switching area. Unexpectedly to our presumption, the AgNPs + Hg^2+^ led to a lower performance that could have occurred due to a higher rate of etching of AgNPs and deposition by Hg ions at higher concentration of 1000 ppm.

### Test of the sensitivity of memristor-based detection of Hg^2+^ real water sample

Since Hg^2+^ at 1000 ppm did not improve the I-V switching behavior, it was worth investigating if I-V switching behavior of AgNPs changes upon the addition of Hg^2+^ at the lower concentration range of 0.1–10000 ppb (Fig. 6c). The measurements in this range of Hg^2+^ clearly indicated that the memristive switching response was linearly improved as the concentration of Hg^2+^was increased from 0.1 to 10000 ppb (Fig. 6c). The results clearly demonstrated that the memristive like behavior of AgNPs was highly selective towards Hg^2+^, therefore, it was used for selective and sensitive detection of Hg^2+^ using the memristive switching principle. The test of the sensitivity of Hg^2+^ detection in spiked SDDW samples in the range of 0.1–10000 ppb indicated 8.7 ppb as the LOD and 26.4 ppb as LOQ (Fig. 6d).

### Study of accuracy and precision of memristor-based detection of Hg^2+^

For the study of accuracy and precision of AgNPs based memristor assay, tap water and SDDW samples spiked with Hg^2+^ in the range of 2.5–7 ppm were analyzed to estimate the intra-day and inter-day recoveries with % RSD. Using this assay, Hg^2+^ in SDDW was recovered intra-day in the range of 66.66–96% with %RSD in the range of 0.005–0.82 and inter-day in the range of 66.66–200% with %RSD in the range of 0.003–0.008. Likewise, the intra-day % recoveries of Hg^2+^ in tap water samples were in the range of 85.3–200% with %RSD in the range of 0.29–0.004 and inter-day in the range of 66.66–104% with %RSD in the range of 0.0041–0.42. In comparison, the accuracy and precision of demonstrated assay were found more suitable for the quantitative determination of Hg^2+^ in SDDW than tap water. This observed difference could have occurred due to the presence of other dissolved impurities in tap water used for preparing spiked test samples. Nonetheless, this study of accuracy and precision indicated that AgNPs based memristor assay for quantitative detection of Hg^2+^ was accurate, precise enough and complied with the standards laid by United States Pharmacopeia (USP)^30,31^.

Previous few studies by various workers had successfully employed AuNPs and AgNPs for selective and sensitive colorimetric detection of Hg^2+^ based on different mechanisms. Chen and coworkers (2013) employed AgNPs of nanoprisms like morphology for highly sensitive and selective colorimetric sensing of Hg^2+^. These authors demonstrated that colorimetric sensing of Hg^2+^ was based on the morphology transition of Ag nanoprisms^37^. In another study, a novel colorimetric nanosensor strategy based on the analyte-induced aggregation of AuNPs for mercury speciation was proposed and demonstrated^38^; whereas different mechanisms such as anti-aggregation of AuNPs^39^ and stabilization of AuNPs by redox-regulated surface chemistry (metal coating) in the presence of ascorbic acid^40^ were successfully implicated and demonstrated. Our study is significantly different from these previous reports as we demonstrated that the selective interaction between phytomediated anionic AgNPs and Hg^2+^ leads to amalgam formation that enables highly sensitive, colorimetric and memristor-based sensing of Hg^2+^.

## Conclusion

In summary, we successfully employed Kokum fruit extract for the biogenic synthesis of unmodified, monodispersed, anionic AgNPs which selectively interacted with Hg^2+^ leading to amalgam formation. The amalgam formed through etching and under potential deposition resulted in a visible color change, change in SPR intensity and also change in memristive switching like property of AgNPs. These changed properties were then subsequently demonstrated for their application in the development of highly sensitive, precise and accurate colorimetric and memristor-based assays for the detection of Hg^2+^ in the water samples.

## Material and Methods

### Materials

All chemicals used in this study were of analytical grade and used without further purification. The stocks and working solutions were prepared in SDDW. Silver nitrate (AgNO_3_) and different cations used were from sulfates or chloride salts. These included cobalt sulphate heptahydrate (CoSO_4_.7H_2_O), barium hydroxide octahydrate (Ba(OH)_2_.8H_2_O), potassium tetra-chloroplatinate (K_2_PtCl_4_), molybdenum trioxide (MoO_3_), mercuric chloride (HgCl_2_), zirconium oxychloride (ZrOCl_2_.8H_2_O), titanium dioxide (TiO_2_), magnesium sulfate heptahydrate (MgSO_4_.7H_2_O), manganese sulphate monohydrate (MnSO_4_.H_2_O), cadmium chloride (CdCl_2_.H_2_O), ferrous sulfate heptahydrate (FeSO_4_.7H_2_O), copper sulfate pentahydrate (CuSO_4_.5H_2_O), lithium perchlorate (LiClO_4_), potassium sulphate (K_2_SO_4_), sodium chloride (NaCl), nickel chloride (NiCl_2_), zinc sulphate (ZnSO_4_.H_2_O), calcium chloride dehydrate (CaCl_2_H_4_O_4_), aluminum chloride(AlCl_2_), chromium trichloridehexahydrate (CrCl_3_.6H_2_O) etc. All metal salts were purchased from Sigma Aldrich (Mumbai, India) and Hi-Media (Mumbai, India). Stock solutions of metal salts were prepared in SDDW and stored at room temperature for further use. All glassware were autoclaved and rinsed with SDDW before use.

### Biosynthesis and preparation of monodispersed AgNPs

The biogenic synthesis of colloidal AgNPs was done using Kokum (*Garcinia indica*) fruit extract as described previously^41^ with slight modifications and used to prepare monodispersed AgNPs by employing differential centrifugation. To this end, initial colloidal AgNPs preparation was first centrifuged at 5000 rpm for 5 min to separate AgNPs pellet (pellet-1) from the supernatant (supernatant-1). Supernatant-1 was then centrifuged at 10000 rpm for 10 min to separate pellet-2 and supernatant-2. Next, supernatant-2 was centrifuged at 15000 rpm for 15 min to get pellet-3 and supernatant-3. Finally, supernatant-3 was centrifuged at 20000 rpm for 20 min to prepare pellet-4 and supernatant-4. All 4 pellets were then washed with SDDW, dispersed in 50 mL SDDW to obtained monodispersed AgNPs and stored at room temperature (RT) for further uses.

### Characterization

Confirmation of biogenic AgNPs was carried out using different spectroscopic and imaging techniques as described previously with slight modifications^41^. To accomplish this, briefly, UV-vis spectroscopy characterization was performed in the wavelength range of 250–800 nm on Bio-Spectrometer (Eppendorf, Hamburg, Germany). The FTIR spectroscopy analysis was performed for both, fruit extract and AgNPs for identification of functional groups from extract involved in the biosynthesis of AgNPs on FTIR spectrometer (Shimadzu, Japan). To obtain a good signal to noise ratio, multiple scans of the AgNPs powder were taken in the range of 500–4000 cm^−1^. The size of AgNPs was determined by DLS and surface charge was determined using PALS zeta potential analyzer Var. 5.76 (Brookhaven Instrument Corp., Holtsville, New York, USA). The elemental composition was detected EDS using x-act with INCA and AZtec EDS analysis software (Oxford Instruments, UK). To study the crystalline nature of AgNPs, the XRD pattern was recorded on XRD (Bruker AXS Analytical Instruments Pvt. Ltd., Germany). The size and morphology of AgNPs were imaged using TEM on Philips TEM CM200 (USA) operating at an acceleration voltage of 20–200 kV. The elemental distribution in complex/amalgam formed between AgNPs and the metal analyte in the selective colorimetric detection reaction was investigated and confirmed by recording the TEM image on JEOL’s JEM UHR-FEG-TEM, 2100 F operating at 200 kV. Also, HAADF-STEM images and EDS spectrum were obtained using the same instrument.

### Screening of metallic cations for the test of selectivity of colorimetric detection

To assess the selectivity of biogenic AgNPs for detection of metals, 20 different metallic cations such as Na^2+^, K^+^, Mg^2+^,Ca^2+^,Ba^2+^, Zn^3+^, Cu^2+^, Ni^2+^, Cd^2+^, Hg^2+^, Co^2+^, Mn^2+^, Pb^2+^, Fe^3+^, Cr^3+^, Zr^3+^, Mo^3+^,Pt^3+^, Li^2+^, Al^3+^ etc were screened to study their interaction with AgNPs. Briefly, metal salt (1000 ppm) solution was added in AgNPs colloidal solution in ratio 1:1 (v/v) and mixed thoroughly, observed for visual change in color or color disappearance and, then monitored for the changes in SPR intensities and spectral shifts by UV–vis spectroscopy in the range of 300–800 nm.

### Colorimetric detection of Hg^2+^ real water samples

AgNPs mediated colorimetric detection of Hg^2+^ was carried out by employing previously demonstrated method^28^ with few modifications. To ascertain the visual limit of detection (LOD) of Hg^2+^, SDDW and tap water samples spiked with Hg^2+^ in the range of 20–200 ppm were mixed thoroughly with AgNPs solution at 1:1 ratio for 5 min. A control sample containing SDDW instead of Hg^2+^ was also prepared and used. All the detection reactions were monitored and observed for visible color change or disappearance while the spectral shifts and changes in SPR intensities were measured using UV-vis. spectrophotometer. For determination of LOD and limit of quantification (LOQ), a standard plot of Hg^2+^concentration against absorbance difference (Δabs) was plotted; a trend line was added to obtain the equation of the straight line and regression value. The LOD and LOQ were then expressed as the lowest concentration of Hg^2+^ that could be detected but not necessarily quantified whereas, the LOQ was estimated as the lowest concentration of an Hg^2+^ that could be determined with acceptable precision and accuracy under the standard conditions.

### Test of accuracy and precision of colorimetric detection of Hg^2+^

To enhance the sensitivity and LOD, the original AgNPs stock solution was diluted five times and used for the preparation of the calibration curve in the range of 0.1–10000 ppb. To accomplish this, SDDW samples spiked with Hg^2+^ in the range of 0.1–10000 ppb were added in AgNPs solution at a 1:1 ratio, mixed thoroughly for 5 min, monitored visually for color change and spectrophotometrically for change in SPR intensities and spectral shifts. The standard graph and linear regression equation with the coefficient were obtained as described above. Using these graphs and equations, the accuracy and precision of colorimetric detection of Hg^2+^ were studied by determining % recovery, intra-day repeatability, and inter-day reproducibility. To this end, test samples were prepared by spiking SDDW and tap water with 2.5, 5 and 7.5 ppm Hg^2+^ and used. Fifty microlitres of each of these test samples were added to 50 µL of AgNPs solution, observed for visible color change or disappearance, change in SPR intensities and spectral shifts. Using the standard graph of Hg^2+^concentration Vs Δabs, Hg^2+^ concentrations in test samples were estimated and compared with original spiked concentration to determine % recovery. For determination of intra-day repeatability and inter-day reproducibility, Hg^2+^ in spiked samples were quantified at three different times in a single day (intra-day) and on three consecutive days (inter-day), respectively. As a measure of accuracy and precision, the percentage relative standard deviations (%RSDs) were then calculated for intra-day repeatability and inter-day reproducibility.

### Fabrication of memristor-based detection device

For fabrication of a memristor-based detection device, a single hole (3 mm in diameter) was bored through fluorine-doped tin oxide (FTO) substrate and other FTO substrate (1 × 3 cm) was placed face to face to form a sandwich structure. Thermo-surlyn (60 μm thick) was then used to bind the two FTO substrates. In this process, the conducting sides of FTO substrates were kept facing each other. Subsequently, the device was heated at 100 °C for 5 minutes to seal the sandwich structure. In this typical sandwich structure, conducting sides worked as metal and space (~60 μm) between them worked as an active layer. The samples to be tested for memristor-based I-V switching behavior were filled in the active layer to perform the electrical measurements using a memristor characterization system (ArC ONE, United Kingdom). Initially, to study the memristive like property of AgNPs and find out the best-suited frequency for good memristive switching, 20 µL of AgNPs was loaded in the active layer of a detection device to perform the electrical measurements at various frequencies of 0.16, 0.8, 4 and 8 Hz. Later, to investigate how the memristive like the behavior of AgNPs responds in presence of Hg^2+^, I-V switching of Hg^2+^ (1000 ppm) alone and AgNPs + Hg^2+^ (1000 ppm) were recorded and analyzed at best suited lowest frequency.

### Test of the sensitivity of memristor-based detection of Hg^2+^ real water sample

The test of the sensitivity of memristor-based detection of Hg^2+^ was studied by estimating the LOD and LOQ. To accomplish this, 10 µL each of SDDW and tap water samples spiked with Hg^2+^ ions in the range of 1–10000 ppb was mixed with 10 µL of AgNPs solution, then loaded in the active detection device for I-V switching measurements. A control sample comprising of 10 µL of SDDW and 10 µL of AgNPs solution was also prepared and used. For the determination of LOD, the standard plot of negative current against concentrations was plotted and used. The LOD and LOQ were determined and expressed as indicated above.

### Study of accuracy and precision of memristor-based detection of Hg^2+^

For the study of accuracy and precision of memristor-based detection, % recovery, intra-day repeatability and inter-day reproducibility were tested. To this end, 10 µL of each of SDDW and tap water samples spiked with 2.5, 5 and 7.5 ppm. Hg^2+^ were mixed with 10 µL of colloidal AgNPs and then loaded in detection device to measure memristive switching behavior (negative current). Using the standard plot of Hg^2+^ concentration against the negative current, the concentration of Hg^2+^ in the test samples was estimated and compared with the original spiked concentrations to determine the % recoveries. As a measure of accuracy and precision, the intra-day repeatability and inter-day reproducibility with %RSDs were determined as indicated above.

## Supplementary information

Supplementary info.
