# Supplementary material for: Selective interaction between phytomediated anionic silver nanoparticles and mercury leading to amalgam formation enables highly sensitive, colorimetric and memristor-based detection of mercury
Source: Sci Rep. 2020 Feb 6;10:2037. doi: 10.1038/s41598-020-58844-4 (PMC7005151; doi:10.1038/s41598-020-58844-4)
Supplement: Supplementary file 1 — Supplementary info. [file 41598_2020_58844_MOESM1_ESM.docx]

**Electronic Supporting Material 
On the ‘Scientific Reports’publicationEntitled**

**“Selective interaction between phytomediated anionic silver nanoparticles and mercury leading to amalgam formation enables highly sensitive, colorimetric and memristor-based detection of mercury”**

**GeetanjaliM. Sangaonkar^1^,Megha P. Desai^1^, Tukaram D. Dongale^1^ and Kiran D. Pawar^1*^**

**^1^School of Nanoscience and Biotechnology, Shivaji University, Kolhapur, Maharashtra, India**

**Figures legends**

**Figure S1**(a)DLS of re-suspended pellet; (b) pellet 2; (c)pellet 3; (d)pellet 4; (e)showing average hydrodynamic size of AgNPs and Zeta potential of biogenic AgNPs of pellet 3.

**Figure S2 S3**FTIR spectrum of Ag-Hg amalgam

**Figure** Sketch of Memristor detection device fabricated and used

**Figures**

**
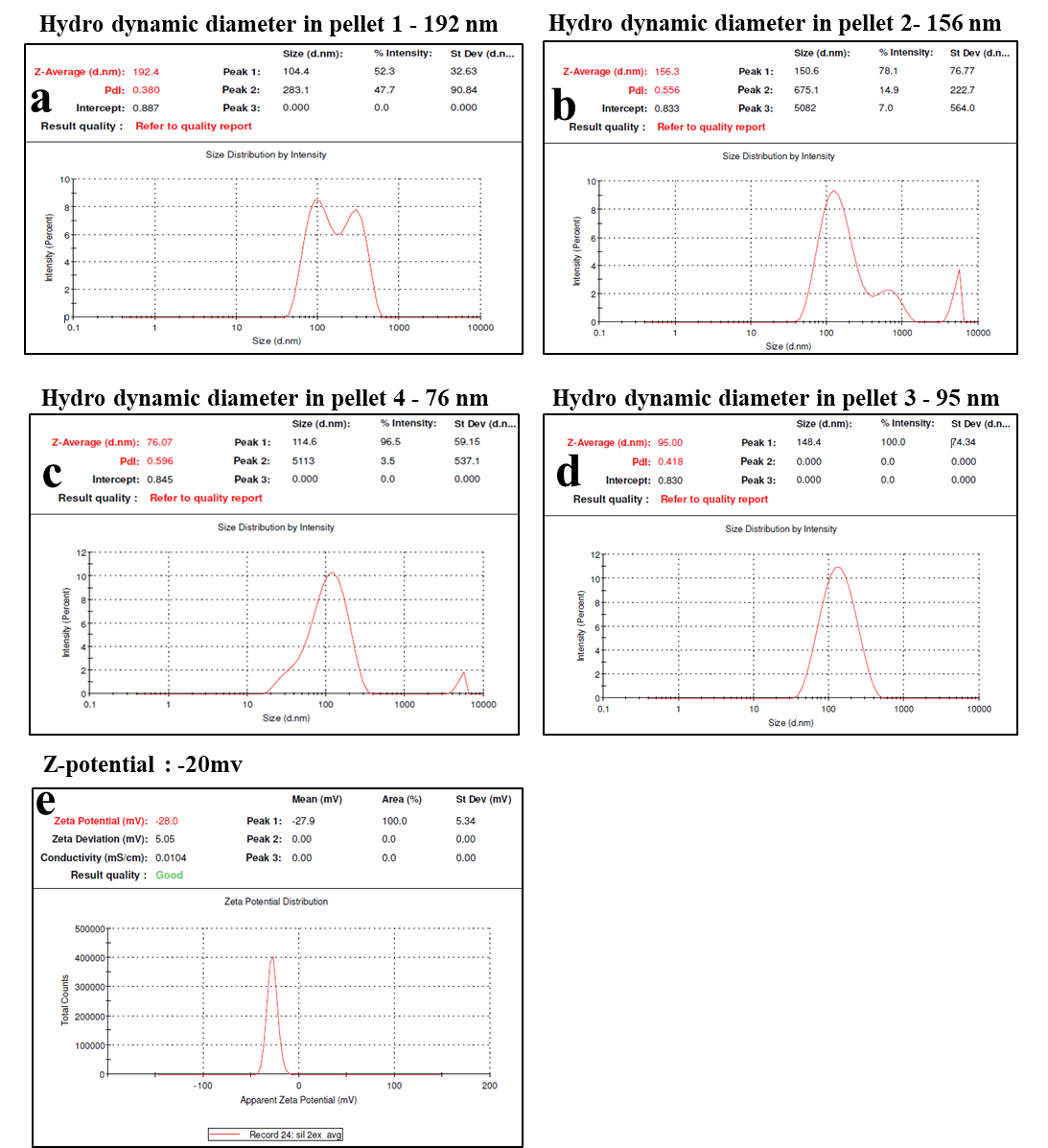
**

**Fig. S1**

**
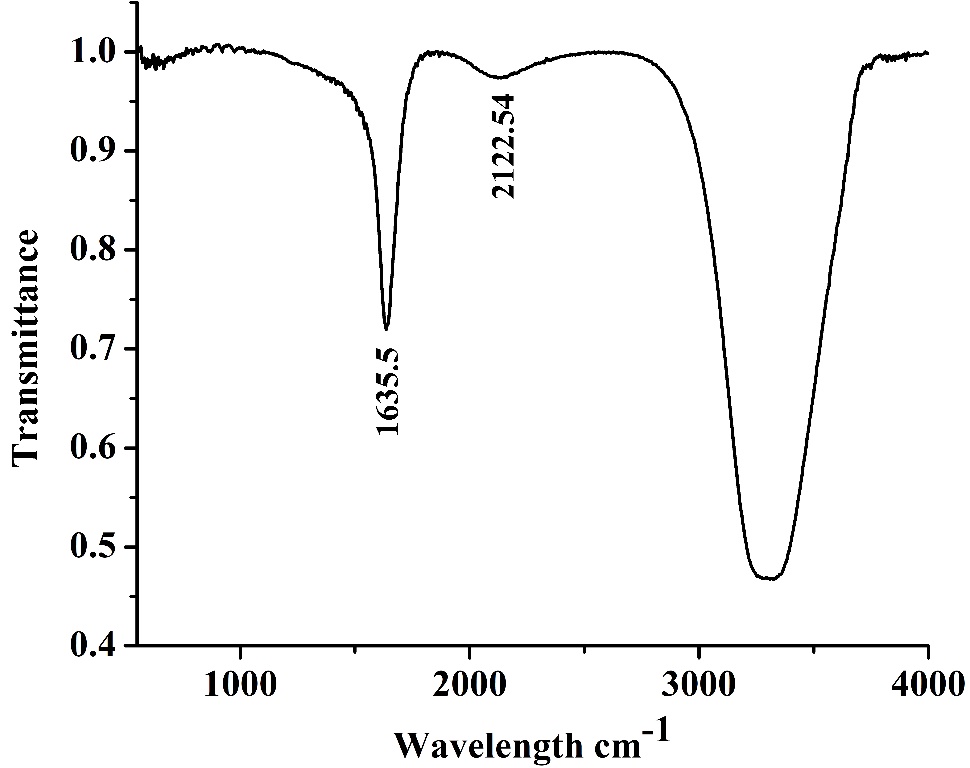
**

**Fig. S2**


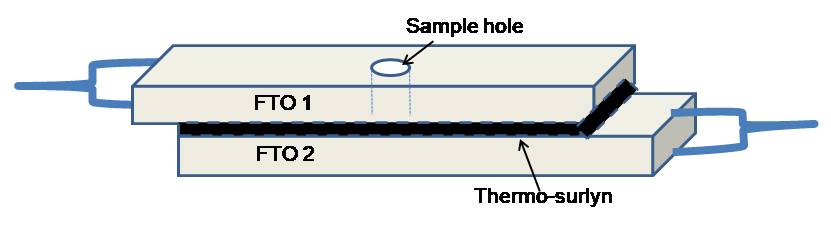


**Figure S3**

**Tables**

**Table S1**Reports on sensitivity of biogenic AgNPs probe based colorimetric detection of Hg^2+^

| **Method** | **Probe** | **LOD/M** | **Reference** |
| --- | --- | --- | --- |
| Uv-vis. spectrometry | Biological AgNPs | 2.2 × 10^-6^ | K. Farhadi et al., 2012 |
|  |  | 8.0 × 10^-7^ | S. Bothra et al., 2013 |
|  |  | 5.0 × 10^-8^ | Rastogi et al., 2014 |
|  |  | 9.38 ×10^-5^ | Ran An., 2016 |
|  |  | 8.5 × 10^-5^ | L. Firdaus et al., 2017 |
|  |  | 3.0× 10^-8^ | Present study 2019 |

**Table S2** Accuracy and precision ofbiogenicAgNPs probe based colorimetric detection of Hg^2+^ in spiked SDDW and tap water samples

| Method | Spiked conc.  (ppm) | Estimated conc.  ( SDDW) | % Recovery (SDDW) | % RSD | Estimated  conc.  ( Tap water) | % Recovery  tap water | %RSD |
| --- | --- | --- | --- | --- | --- | --- | --- |
|  | 2.5 | 2.77 | 110.8 | 0.44 | 2.88 | 115.2 | 0.73 |
| Intra-day | 5 | 4.6 | 98.2 | 0.11 | 5.48 | 109.6 | 0.33 |
|  | 7.5 | 8.03 | 107.6 | 0.26 | 7.18 | 95.73 | 0.36 |
|  | 2.5 | 2.83 | 113.2 | 0.41 | 2.96 | 118.4 | 0.89 |
| Inter-day | 5 | 5.2 | 104 | 0.85 | 5.48 | 109.6 | 0.65 |
|  | 7.5 | 7.96 | 106 | 0.02 | 7.51 | 100.1 | 0.15 |
